# Searching for Value Sensitive Design in Applied Health AI: A Narrative Review

**DOI:** 10.1055/s-0044-1800723

**Published:** 2025-04-08

**Authors:** Yufei Long, Laurie Novak, Colin G. Walsh

**Affiliations:** Department of Biomedical Informatics, Vanderbilt University Medical Center, Nashville, TN

**Keywords:** Value-sensitive design, Artificial Intelligence, Ethical value – Universal Design, Healthcare

## Abstract

**Objective** : Recent advances in the implementation of healthcare artificial intelligence (AI) have drawn attention toward design methods to address the impacts on workflow. Lesser known than human-centered design, Value Sensitive Design (VSD) is an established framework integrating values into conceptual, technical, and empirical investigations of technology. We sought to study the current state of the literature intersecting elements of VSD with practical applications of healthcare AI.

**Methods** : Using a modified VSD framework attentive to AI-specific values, we conducted a narrative review informed by PRISMA guidelines and assessed VSD elements across design and implementation case studies.

**Results** : Our search produced 819 articles that went through multiple rounds of review. Nine studies qualified for full-text review. Most of the studies focused on values for the individual or professional practice such as trust and autonomy. Attention to organizational (e.g., stewardship, employee well-being) and societal (e.g., equity, justice) values was lacking. Studies were primarily from the U.S. and Western Europe.

**Conclusion** : Future design studies might better incorporate components of VSD by considering larger domains, organizational and societal, in value identification and to bridge to design processes that are not just human-centered but value sensitive. The small number of heterogeneous studies underlines the importance of broader studies of elements of VSD to inform healthcare AI in practice.

## 1. Introduction

Value Sensitive Design (VSD) represents a comprehensive approach to embed human values in the creation of technology. A topical and timely area of healthcare innovation, artificial intelligence (AI) describes the use of computers and technology to simulate intelligent behavior comparable to human critical thinking [ 1 ]. Public perception of recent advances in AI and widespread availability of AI-based tools have catalyzed interest in their clinical application [ 2 3 4 5 6 ]. Current applications of AI in healthcare include medical imaging and diagnostics, personalized medicine, virtual health assistants and chatbots, and predictive analytics and risk assessment [ 7 ]. These applications rely on computational methods ranging from machine learning (ML), natural language processing (NLP), computer vision (CV), and generative models such as large language models (LLMs).

Healthcare AI has been a mainstay of predictive and preventive clinical decision support (CDS) for decades and has been the subject of rigorous evaluation [ 9 ]. Scalable algorithmic tools present the promise of dramatically improving care while also risking the perils of harm at scale [ 10 ]. Creating and deploying AI without robust ethical frameworks and rigorous testing might perpetuate bias, discrimination, and introduce privacy, security risks [ 11 ]. Regardless of the nature of underlying AI, generative or not, actionable healthcare AI requires careful integration of algorithmic insights into clinical workflow, which can be achieved through effective design that engages patients and healthcare professionals, including clinicians, nurses, and allied health staff.

Numerous views of design have emerged over the last fifty years [ 12 ]. Design disciplines emanate from different industries and areas, such as human factors and interaction design linked to experimental psychology, and design thinking which is rooted in the science of creativity [ 12 ] More recently in healthcare, human-centered design (HCD) has emerged as a means of achieving effective design and lessening potential for risk [ 13 14 15 ]. In parallel, concepts like algorithmovigilance for healthcare AI have broadened the discussion around preventing adverse effects of implemented AI [ 16 ]. VSD intends to place human values at the heart of technological design. Batya Friedman *et al.* , first introduced VSD in the 1990s, challenging the prevailing notion of technology as value-neutral or simply a tool for human activities. Per Friedman *et al.* , values are defined as “what is important to people in their lives, with a focus on ethics and morality” [ 13 ]. Commonly recognized examples of such values include autonomy, trust, equity, and justice. The VSD framework has been applied across various fields and has become a recognized approach for addressing values in technological design [ 17 ]. While sharing some similarities with HCD in terms of considering users' needs and experiences, VSD might complement HCD by considering social and ethical implications of technology and impacts on various stakeholders and society at large. The framework emphasizes three main areas of investigation: conceptual, empirical, and technical. Each area builds on the prior, with conceptual investigations articulating values and any value conflicts that might result from technology use. Empirical investigations apply qualitative and quantitative methods to crystallize and document stakeholders' values, needs, and practices. Finally, technical investigations focus on the design and features of the technology itself, examining how values are or can be supported.

We emphasize the importance of applying VSD to healthcare AI because of the potentially rapid development cycle, scalable nature, lack of knowledge among healthcare managers about how it works, and its potential societal impact. The accelerated pace of AI advancement has prompted ongoing debate concerning oversight and regulation. Government entities, professional societies, scientific leadership groups, and other organizations worldwide have begun issuing principles and guidance to attempt to outline and mitigate large-scale societal risks [ 18 19 20 21 ]. Such concerns underline the need to characterize the societal values that should be represented in the legislation and professional standards that will undoubtedly emerge. Similarly, while HCD incorporates numerous strengths in the pursuit of effective AI tools, a recent systematic review suggests key sociotechnical gaps remain: technical limitations ( *e.g.* , predicted AI output will be inaccurate), workflow misalignment ( *e.g.* , AI tools will add to workload), attitudinal barriers ( *e.g.* , resistance to using AI tools, lack of desire to use AI tools), informational barriers ( *e.g.* , AI output lacks actionable information to make the relevant clinical decision), usability issues ( *e.g.* , lengthy ‘click-throughs’ as inputs to use AI tools), and environmental barriers ( *e.g.* , requirements of AI tools such as high quality retinal imagery not available in routine practice) [ 9 ]. Many of these gaps might be lessened by design that centers not just on the performance of the humans interacting with the technology, but also on their values and the values of other relevant stakeholders.

To better understand how well values are captured in modern healthcare AI, we conducted a narrative review of the literature intersecting VSD, AI, and the design and implementation of AI-driven healthcare technology. We assessed recent literature, 2022 through 2023 against Friedman's tripartite framework including modifications proposed by Umbrello *et al.* , incorporating Artificial Intelligence for Social Good (AI4SG) [ 17 , 22 ]. We then evaluated all eligible studies for the presence of elements of VSD, critically doing so regardless of whether VSD was directly acknowledged, as it is a newer and lesser-known concept in the field than HCD. By demonstrating and dissecting VSD methodology through real-world case studies, we aim to highlight areas of strength and potential directions for improvement in the design of AI-driven technology that is both human-centered and value-sensitive.

## 2. Methods

We conducted a narrative review informed by the PRISMA guidelines for systematic reviews [ 23 ] with expectation that the state of the literature would not permit meta-analysis. We searched PubMed, using a search query representing AI, design, and value. The PubMed search query is shown (Box 1).

The initial list of publications was reviewed via title and abstract review by two authors (Y.L., L.N.) to establish a full-text candidate list. The authors reviewed initial results to eliminate papers that did not address a healthcare area and/or were not English-language. Systematic reviews were also excluded though they informed study design and background as above. Retracted studies identified by initial search were excluded. The final group of studies was reviewed for elements of VSD and the presence of a specific AI application, tabulated, and summarized with respect to clinical application area, geographic diversity, methods, stakeholder engagement methods, and VSD findings.

## 3. Results

**Figure 1.**
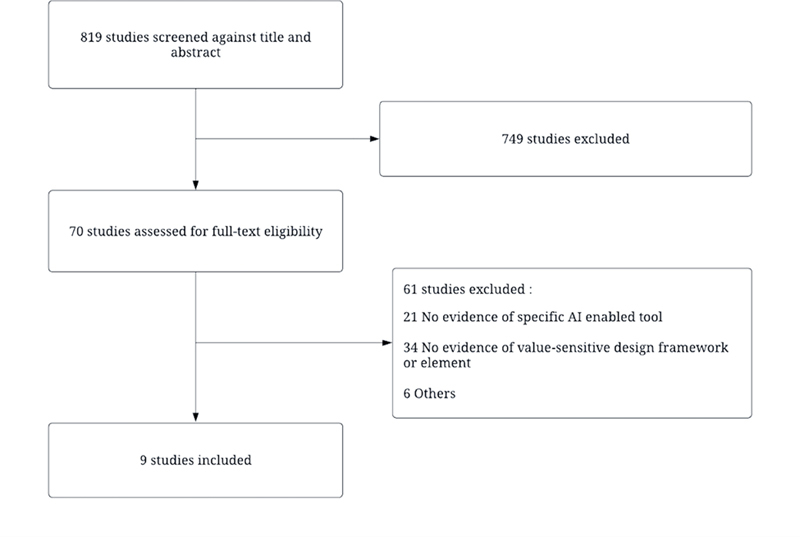
Flow diagram of the search strategy.

From 2022 through January 2024, we identified 819 publications via the initial search; five of the 819 had been retracted and were excluded. The initial reviews resulted in 70 studies requiring full-text consideration and nine (9) studies being selected for full-text review and extraction. A flow diagram summarizing the search is shown ( Figure 1 ).

Table 1 summarizes all studies reviewed in full-text including study objective and key findings relevant to our survey. In the final group of studies, most were original research or case studies. Clinical application area of these studies varied widely from rare disease [ 24 ], dementia [ 25 ], sexual health [ 26 ], mental health [ 27 ], to stress management [ 28 ]. No two studies that met full text eligibility were in the same clinical domain. In terms of AI application area, use cases primarily described predictive model and CDS or consumer health informatics applications with mobile devices (smartphones).

**Table 1.** Summary of all studies reviewed.

Article	Stakeholders	Sample Size	Study Objective	Major Findings	Examples of Value Discussed	AI Methods Studied
Cagliero et al. (2023)	Patients, clinicians, AI designers	17	Evaluate a „values collision“ approach to identify ethical challenges associated with ML applications to healthcare (ML-HCA) for advance care planning (ACP).	Five key „values collisions“ were identified between stakeholders (patients, clinicians, designers) regarding the ML-enabled ACP application, marking areas of ethical tension ranging from end-of life workflow, which stakeholders receive prediction, to how and if to protect early deployment research. From these findings, the design team prioritized examining alternative workflow strategies, clarifying that mortality prediction was only to identify ACP need (not other mortality-related ends), and shielding ongoing research from social media scrutiny.	Article showed value collisions between stakeholders by different ethical concerns including bias, transparency, patient consent, and patient involvement.	Supervised machine learning (tree-based risk models) to predict patient mortality
Cenci et al. (2023)	Danish patients with dementia (referred as „citizens“ in the manuscript), relatives, volunteers, local authorities, dementia coordinators, healthcare professionals, AI/data science experts, and social scientists/philosophers)	41	Demonstrate how a participatory value-sensitive design approach can be used to develop an AI-based tracking smartphone (mHealth) app targeting Danish citizens with dementia.	This illustrative case study explored value construction and elicitation, demonstated how moral dilemmas and value conflicts result from diverse needs or vested interests can be resolved in practice. Through interations of focus group-based deliberative workshops, both expert and non-expert stakeholders including those with dementia directly contributed to identifying values to guide the app's design to be more ethical and democratic.	Safety, privacy, social support	Real-time anomaly detection using spatiotemporal data
Fischer et al. (2023)	Obstetrician-gynecologists (OB-GYNs)	13	Explore obstetricians' perceptions about the potential value of AI in clinical practice.	Perceived usefulness and trust are two major themes identified from the interviews. The result show 2 paradoxes between what the clinicians found most valuable in an AI model and and what they considered necessary to trust an AI model. That is, AI is expected to provide added value by surpassing human capabilities but there is a great need for explainability. Additionally, participants desire comprehensive models with many parameters, yet acknowledged that certain contextual factors should only be considered by humans	Trust	AI scenarios: diagnosis of intrapartum fetal asphyxia, hospital discharge of women with pre-term labor, and personalized treatment for gestational diabetes.
Götzl et al. (2022)	Young people and experts (psychologists, educators, respresentives of the digital industry)	Focus group (n=8) Expert interviews (n=5) Online survey (n=666)	Explore the subjective needs, attitudes, and preferences of key stakeholders (young people and experts) towards an AI-informed mobile mental health app (mHealth).	Both experts and young people showed general acceptance and interest towards AI-informed mHealth. Young people showed openness to AI and data sharing, at the same time demanded transparency and control over personalization. Experts perceived AI-informed mHealth apps as opportunities to complement on-site delivery of interventions, improving the chance to reach at-risk youth.	Transparency, trust	mHealth app using ecological momentary assessment (EMA) data.
Hallowell et al. (2022)	Clinical geneticists, data scientists, bioinformaticians, industry and patient support group spokespersons	20	Explore under what conditions medical AI tools that employ machine learning can be trusted, using the case of computational phenotyping (CP) to support diagnosis of rare disease.	Interviewees emphasized the importance of establishing trust in the use of computational phenotyping tools in identifying rare disease. Interviewees discussed trust both in the context of relational trust, trusting those who use and develop AI, and epistemic trust, trusting in the knowledge produced by the AI technology. Both patients' trust for clinicians and clinicians' trust for AI developers were discussed. Trust in the technology itself requires demonstrating reliability and accuracy of algorithm output and involving trusted individuals in using/developing the tools.	Trust	Computational phenotyping
Helenason et al. (2023)	Primary care physicians (PCPs)	Interviews (n=15) Diagnostic assessment on image interpretation (n=25)	Employ qualitative and quantitative methods to investigate the feasibility of an AI-based clinical decision support system (CDSS) to detect cutaneous melanoma in primary care.	Interviews with primary care physicians revealed that trust is a central concern, which can be improved by evidence of sufficient diagnostic accuracy. „Usability and user experience“ and „the clinical context“ also emerged as qualitative themes. When assessing images, adding AI decision support increased physicians' sensitivity and diagnostic accuracy compared to their assessments without the use of AI.	Trust	Convolutional neural network for dermatological image analysis
Helman et al. (2023)	Intensive Care Unit (ICU) clinicians (nurses, physician assistants, physicians)	23	Engage multidisciplinary intensive care unit (ICU) users (physicians, nurse practitioners, physician assistants) in the development of a graphical user interface (GUI) to present an AI-derived patient instability risk score.	Six themes emerged: analytics transparency, graphical interpretability, impact on practice, value of trend synthesis of dynamic patient data, decisional weight, and display location. All disciplines valued synthesized views but were skeptical of heavily weighing AI output until proven trustworthy.	Autonomy, transparency, trust	Predictive modeling, clinical decision support
Kerr et al. (2023)	Employees of a Swiss insurance company	170	Identify relevant values and assess potential users' comprehension of these values to derive requirements for the value sensitive design of a digital health solution for workplace stress. This digital stress management intervention at the workplace relies on ML to continuously predict the user's current stress level using multiple data modalities: physiological, behavioral, and contextual data sources.	The values health and well-being, privacy, autonomy, accountability, and identity (defined as people's understanding of who they are over time) were identified through literature search. Employees showed moderate to high intention to use a digital stress management intervention but had concerns regarding effectiveness and privacy. Privacy and accountability concerns are higher when machine learning monitoring was involved. Integrability, user-friendliness, and digital independence emerged as novel values from the qualitative analysis.	Health and well-being, privacy, autonomy, accountability, identity	Real-time predictive modeling
Wang et al. (2022)	User of SnehAI (AI chatbot) application: young people in India	Qualitative data: 2 in-person group meetings, 4 virtual conference, unspecified number of participants; Quantitative data: 135, 263 unique chatbot users (8.2 million messages exchanged)	Use Gibson's theory of affordances to examine SnehAI, an AI chatbot for sexual and reproductive health, and offer guidance on how such chatbots can promote sexual and reproductive health and advocate for health entitlements of women and girls in India. Affordances serve as a mechanism to identify values in this case.	SnehAI demonstrated strong evidence across fifteen functional affordances, several of them reflecting social values: accessibility, multimodality, nonlinearity, compellability, queriosity, editability, visibility, interactivity, customizability, trackability, scalability, glocalizability, inclusivity, connectivity, and actionability. SnehAi effectively engaged its users, especially young men. SnehAI presented itself as a trusted source of educational and entertaining information and its natural language processing system worked effectively to personalize the response and optimize user experience.	Equitable access, civility, inclusivity	NLP Chatbot

### 3.1. Stakeholder Engagement and Methods

Unlike many AI-driven CDS studies aimed at clinician-users, studies in this survey engaged a wide range of stakeholders from the general public, employees in the workplace, patients with lived experience, clinicians, managers, and biomedical scientists. Geographic diversity was limited, however, with the studies set solely in European countries and the United States. Methods used included interviews (three studies), focus groups (two studies), participatory design workshops (two studies), surveys (two studies), and literature review (one study).

### 3.2. VSD Themes Addressed

The predominant element of VSD addressed in six of nine included studies was that of value identification. Two addressed context analysis and the same two addressed prototyping.

### 3.3. Studies incorporating methods of VSD

Cagliero *et al.* , [ 29 ] explored the values of stakeholders in the context of a ML tool for advanced care planning (ACP) using a values-collision approach that describes the tensions or collisions between various stakeholders' perspectives. Stakeholders included clinicians, designers, administrators, and patients. They found collisions in the values of the stakeholders that related to model characteristics, model implementation, and intervention benefits and harms. The authors propose the values-collision approach as a useful strategy for using tensions as a source of useful input for the design team to make improvements with ethical consequences in mind.

Cenci *et al.* , [ 25 ] used the VSD framework in a citizen science project focused on the design of an mHealth app for dementia patients that involved location surveillance as a feature. The investigators used focus groups to elicit values of stakeholders including citizens with dementia, their family members, clinical staff and volunteers, and leaders of the local smart city initiative. They describe the use of a procedural-deliberative method to engage a wide variety of stakeholders in the co-creation of the ultimate design. Of note, the investigators referred to participants in their study as “citizens” and not patients.

### 3.4. Studies identifying trust as a core value

Götzl *et al.* , [ 27 ] used focus groups, interviews, and a survey to examine the perspectives of key stakeholders of a mobile mental health app. The investigators documented a variety of values and related dimensions including acceptability of specific types of data sharing, perspectives on the role of an app in relation to traditional mental health providers, and the level of trust in various types of institutions.

Hallowell *et al.* , [ 24 ] examined a specific domain, trust, in a study that documented the conditions under which stakeholders felt ML could be trusted in the diagnosis of rare disease. The authors interviewed geneticists, data scientists, bioinformaticians, industry representatives, and patient representatives. Participants characterized relational trust between patients and clinicians as a key element in determining the acceptability of a technology, and expressed both concerns and enthusiasm about the prospect of commercial involvement in the process.

Helenason *et al.* , [ 30 ] engaged primary care physicians in a simulation and interviews that explored feasibility of AI-based CDS to detect cutaneous melanoma. The physicians expressed concerns about trusting the technology, and these concerns converged with issues related to the clinical context, specifically that the providers in this context are responsible for the effective use of clinical guidelines for a wide variety of clinical issues.

Helman *et al.* , [ 31 ] engaged various clinical personnel in focus groups in the development of a graphical user interface (GUI) to display the AI-based risk scores for instability in the intensive care unit (ICU). Stakeholder values that emerged were trust, the impact of the tool on practice and decision-making of novice personnel, and weighing of AI outputs during decision-making.

Fischer *et al.* , [ 32 ] interviewed Dutch obstetric professionals to gain their perspectives on the use of AI in obstetric care using three common AI scenarios: diagnosis, workflow optimization/ triage, and personalized treatment. Two key paradoxes emerged: the first was the desire to know the factors that drive predictive models while acknowledging that models exceed human capacity in pattern identification, and the second was the desire for the model to have access to as many parameters as possible for accuracy, while acknowledging the importance of contextual factors that the model could not capture.

### 3.5. Studies emphasizing organizational or societal values not addressed above

Kerr *et al.* , [ 28 ] used a literature review and survey to examine employee concerns about digital stress management interventions (dSMI), including those with and without ML-based just-in-time adaptive interventions (JITAI). The literature review identified health and well-being as a central value, supporting use of dSMI in the workplace. Employees were concerned about privacy, autonomy, identity, and accountability in relation to the use of such tools.

Wang *et al.* , [ 26 ] use Gibson's concept of affordances [ 33 ] to frame and facilitate a study of features that promote use of a sexual health chatbot among youth, and to explore actual use of the chatbot. An affordance is a feature or resource that assists users in appropriate use of an object. The authors' work resulted in evidence across fifteen affordances they deemed functional (including new ones that emerged in this study), however several of the affordances reflected societal values, such as inclusivity and accessibility. The complete list of affordances included accessibility, multimodality, nonlinearity, compellability, “queriosity” (encouraging user curiosity), editability, visibility, interactivity, customizability, trackability, scalability, localizability (enabling linkages between users and services in their local area), inclusivity, connectivity, and actionability. This study illustrates how values can be derived using existing frameworks for characterizing human interactions with technology.

## 4. Discussion

**Figure 2.**
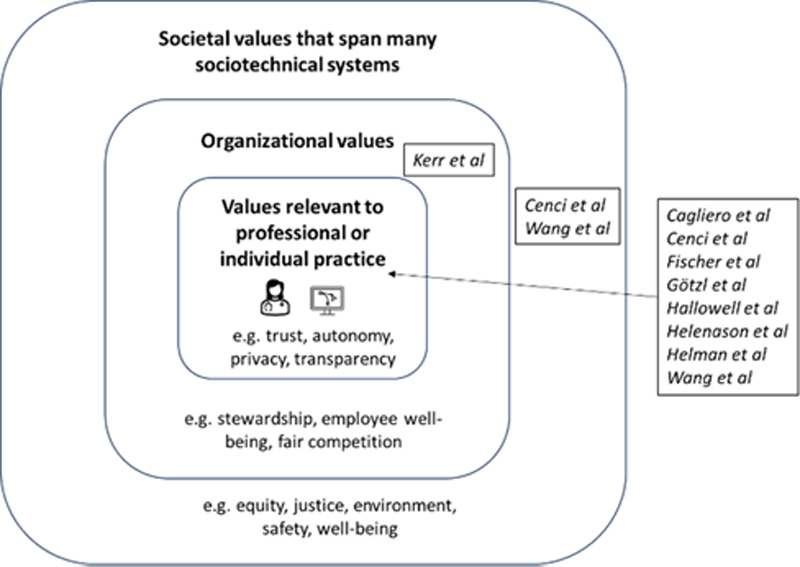
Classification of values identified in the studies.

This narrative review evaluated the recent biomedical literature at the intersection of applied healthcare AI and VSD. We emphasized applied studies and those with design-focused research methods, which led to numerous Empirical investigations per the VSD framework. The literature on design of AI-driven technology largely addressed value identification across heterogeneous clinical domains. VSD concepts are not common topics in healthcare AI, so we assessed for elements of the framework regardless of whether the language of VSD was specifically included in the manuscript. Studies identified were conducted almost entirely in the United States and Western Europe, leaving significant opportunity to broaden similar efforts globally. In sum, we identified a small number of heterogeneous studies emphasizing values relevant to professional or individual practice (see Figure 2 ). While such values are critical to effective healthcare AI, VSD remains understudied in this highly topical domain of biomedical innovation. As such, we cannot yet recommend broad uptake of VSD principles into healthcare AI studies but do advocate for more comprehensive investigations of VSD in future work.

Regarding the specific values identified or explored in the papers, we found that they largely focused on values relevant to professional or individual practice as shown in the inner square of Figure 2 . We found that studies such as Hallowell *et al.* , [ 24 ], Helanason *et al.* [ 30 ], and Kerr *et al.* , [ 28 ] focused on acceptability or usability of technology and were also positioned methodologically to identify such practice-based values related to technology. Organizational values were represented in Kerr *et al.* [ 28 ], where employee well-being was the primary driving force for the project. Societal values were less often elucidated though Wang *et al.* , addressed some in their study of affordances in the context of an educational chatbot.

### 4.1. Implications for AI development and use in healthcare

AI-driven technology focused on individual or practice level values might implicate values for organizations or society. While every applied study cannot comprehensively elucidate and address all possible values, a wider lens on value identification might identify potential gaps that might widen during or after implementation. This approach also might lead to more holistic evaluation of AI-driven clinical technology in practice. For example, clinicians using text generative tools to speed clinical documentation might note impacts of such technology on their autonomy and need for trust. However, such tools also might impact the well-being of those working at the organization as well as broaching concerns around equity depending on how the models were trained, and whether training corpora were representative of the patient population at that organization.

Value identification also might reveal value tensions, which arise when multiple human values that co-exist in a given context come into opposition with one another [ 17 ]. Per Friedman *et al.* [ 17 ], the optimal solution aims to attend to all values intact rather than selecting one over another. Cagliero *et al.* , [ 29 ]provided an illustration of value tensions and the resulting effort to address such tensions. Value tensions can manifest at multiple levels - within individuals attempting to balance competing values internally, between individuals who prioritize values differently, and between entire groups who have contradictory perspectives. As use of AI expands, so will the potential for these tensions and the need for effective means of resolving them.

### 4.2. Strengths and limitations

Strengths of this work include a novel focus on the intersection of AI and VSD. We reviewed recent literature, ensuring the results described are timely and account at least in part for the emergence in recent years of a focus on human centered design in informatics [ 14 ]. We broadened published definitions of VSD based in interpretations by Umbrello *et al.* , [ 22 ]. We also reviewed studies for presence of VSD elements regardless of whether the authors applied VSD directly, as we hypothesized modern HCD would incorporate VSD concepts given the attention to issues such as trust, autonomy, etc., represented with variable verbiage as we found here.

Limitations of this study include an emphasis on applied AI-driven technology design in healthcare, which biased results toward Empirical VSD investigations. The focus on AI, HCD and VSD, might also have led to relevant studies being excluded from further review. Our search was limited to PubMed.

## 5. Conclusions

Elements of VSD have been included in empirical studies of AI-driven technology in healthcare with clear emphases on values to individual and professional practice, especially trust. As users gain trust in such technology, system designers might broaden their design approaches to glean values across organizational and societal dimensions. This attention might inform more effective tools, more comprehensive evaluation, and more impactful design.

## Notes

Box 1. Reproducible Search Query in PubMed. (value sensitive design[tiab] OR value-sensitive design OR value[tiab] OR social value[tiab] OR social good[tiab] OR ethical value[tiab] ) AND (“Universal Design”[Mesh] OR universal design[tiab] OR design[tiab] OR human-centered design[tiab] OR “User-Centered Design”[Mesh] OR user-centered[tiab] OR user-centred[tiab] OR user-centric[tiab] ) AND (“Artificial Intelligence”[Mesh] OR artificial intelligence[tiab] OR AI [tiab] OR intelligent design[tiab] OR intelligence design[tiab] OR machine intelligence[tiab] OR computational intelligence[tiab] OR “Machine Learning”[Mesh] OR machine learning[tiab] OR learning machine[tiab] OR “Deep Learning”[Mesh] OR deep learning[tiab] OR “Natural Language Processing”[Mesh] OR natural language processing[tiab]) AND (2022:2023[pdat])
